# 2-Meth­oxy-4-methyl­phenyl 4-toluene­sulfonate

**DOI:** 10.1107/S1600536808025749

**Published:** 2008-08-16

**Authors:** G. Ramachandran, Charles Christopher Kanakam, B. Gunasekaran, V. Manivannan

**Affiliations:** a Department of Chemistry, Valliammai Engineering College, Chennai, India; b Department of Physics, AMET University, Kanathur, Chennai, India; c Department of Physics, Presidency College, Chennai 600 005, India

## Abstract

In the title mol­ecule, C_15_H_16_O_4_S, the inter­planar angle between the two aromatic rings is 45.07 (7)°. The crystal packing is stabilized by weak inter­molecular C—H⋯O inter­actions.

## Related literature

For related literature, see: Manivannan *et al.* (2005*a* ▶); Spungin *et al.* (1984 ▶); Yachi *et al.* (1989 ▶). Similar compounds have been reported by: Manivannan *et al.* (2005*b* ▶); Ramachandran *et al.*(2007 ▶).
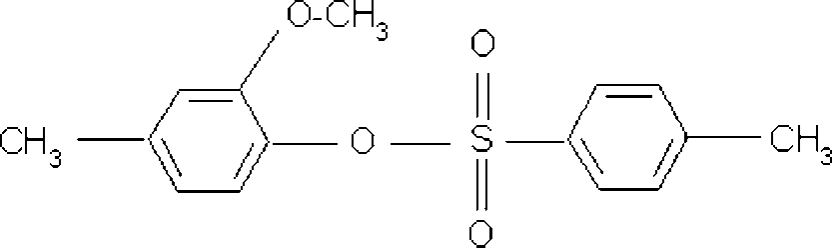


## Experimental

### 

#### Crystal data

C_15_H_16_O_4_S
*M* *_r_* = 292.34
Triclinic, 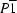

*a* = 7.932 (2) Å
*b* = 8.736 (3) Å
*c* = 10.934 (3) Å
α = 93.785 (5)°
β = 98.453 (5)°
γ = 102.476 (4)°
*V* = 727.9 (4) Å^3^
*Z* = 2
Mo *K*α radiation
μ = 0.23 mm^−1^
*T* = 293 (2) K
0.48 × 0.46 × 0.14 mm

#### Data collection

Bruker Kappa APEXII diffractometer
Absorption correction: multi-scan (*SADABS*; Sheldrick, 1996 ▶) *T*_min_ = 0.902, *T*_max_ = 0.970
8439 measured reflections
3348 independent reflections
2220 reflections with *I* > 2σ(*I*)
*R*_int_ = 0.028

#### Refinement

*R*[*F* ^2^ > 2σ(*F* ^2^)] = 0.053
*wR*(*F* ^2^) = 0.140
*S* = 1.02
3348 reflections
184 parameters
H-atom parameters constrained
Δρ_max_ = 0.30 e Å^−3^
Δρ_min_ = −0.20 e Å^−3^

### 

Data collection: *APEX2* (Bruker, 2004 ▶); cell refinement: *APEX2*; data reduction: *APEX2*; program(s) used to solve structure: *SHELXS97* (Sheldrick, 2008 ▶); program(s) used to refine structure: *SHELXL97* (Sheldrick, 2008 ▶); molecular graphics: *PLATON* (Spek, 2003 ▶); software used to prepare material for publication: *SHELXL97*.

## Supplementary Material

Crystal structure: contains datablocks global, I. DOI: 10.1107/S1600536808025749/bt2764sup1.cif

Structure factors: contains datablocks I. DOI: 10.1107/S1600536808025749/bt2764Isup2.hkl

Additional supplementary materials:  crystallographic information; 3D view; checkCIF report

## Figures and Tables

**Table 1:** Hydrogen-bond geometry (Å, °)

*D*—H⋯*A*	*D*—H	H⋯*A*	*D*⋯*A*	*D*—H⋯*A*
C15—H15*C*⋯O1^i^	0.96	2.60	3.327 (4)	133

Symmetry code: (i) 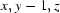.

**Fig. 1.**
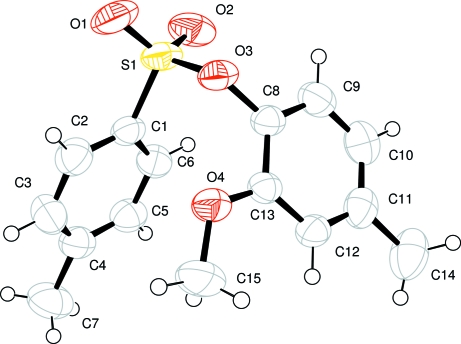
The molecular structure of the title compound with atom labels and 50% probability displacement ellipsoids for non-H atoms.

**Fig. 2.**
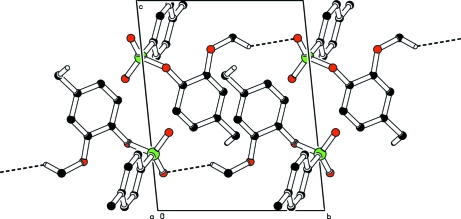
The packing of the title compound viewed down the a axis. Hydrogen bonds are shown as dashed lines. H atoms not involved in hydrogen bonding have been omitted.

## supplementary crystallographic information

### Comment

Several compounds containing the *para*-toluene sulfonate moiety are used in the fields of biology and industry. The merging of lipids can be monitored using a derivative of *para*-toluene sulfonate (Yachi *et al.*, 1989). This method has been used in studying the membrane fusion during the acrosome reaction (Spungin *et al.*, 1984).

The geometric parameters in the title compound agree with the reported values of similar structures (Manivannan *et al.*, 2005*a*,*b*; Ramachandran *et al.*, 2007). The aromatic rings make a dihedral angle of 45.07 (7)° with respect to each other. The crystal packing is stabilized by weak intermolecular C—H···O interactions.

The angle between the two oxygen atoms of the –SO2- group is much larger than the tetrahedral angle which leads to the decrease in the –O—S—Ar angle.

### Experimental

A solution of vanillin in toluene was treated with amalgamated Zinc wool and concentrated HCl under reflux for thirty hours. The reaction mixture was cooled. The organic layer was separated and the aqueous layer was washed with ether. The combined ether and toluene solutions were washed with water, dried over anhydrous calcium chloride and the solvent removed under reduced pressure to get 2-methoxy-4-methyl phenol. A mixture of the above phenol and triethyl amine in acetone was treated with *p*-toluene sulfonyl chloride at room temperature for twelve hours. The residue was washed with triethyl amine solution and water and dried. Diffraction quality crystals were obtained from the solid by recrystallizing from ethanol.

### Refinement

H atoms were positioned geometrically and refined using riding model with C—H = 0.93 Å and *U*_iso_(H) = 1.2Ueq(C) for aromatic C—H, C—H = 0.96 Å and *U*_iso_(H) = 1.5Ueq(C) for CH_3_. The methyl groups were allowed to rotate but not to tip.

### Crystal data

**Table:**

C_15_H_16_O_4_S	*Z* = 2
*M**_r_* = 292.34	*F*_000_ = 308
Triclinic, *P*1	*D*_x_ = 1.334 Mg m^−^^3^
*a* = 7.932 (2) Å	Mo *K*α radiation λ = 0.71073 Å
*b* = 8.736 (3) Å	Cell parameters from 2654 reflections
*c* = 10.934 (3) Å	θ = 1.9–24.7º
α = 93.785 (5)º	µ = 0.23 mm^−^^1^
β = 98.453 (5)º	*T* = 293 (2) K
γ = 102.476 (4)º	Block, colourless
*V* = 727.9 (4) Å^3^	0.48 × 0.46 × 0.14 mm

### Data collection

**Table:**

Bruker KappaAPEX2 diffractometer	3348 independent reflections
Radiation source: fine-focus sealed tube	2220 reflections with *I* > 2σ(*I*)
Monochromator: graphite	*R*_int_ = 0.028
*T* = 293(2) K	θ_max_ = 28.0º
ω and φ scans	θ_min_ = 1.9º
Absorption correction: multi-scan(SADABS; Sheldrick, 1996)	*h* = −10→10
*T*_min_ = 0.902, *T*_max_ = 0.970	*k* = −11→11
8439 measured reflections	*l* = −13→14

### Refinement

**Table:**

Refinement on *F*^2^	Secondary atom site location: difference Fourier map
Least-squares matrix: full	Hydrogen site location: inferred from neighbouring sites
*R*[*F*^2^ > 2σ(*F*^2^)] = 0.053	H-atom parameters constrained
*wR*(*F*^2^) = 0.140	*w* = 1/[σ^2^(*F*_o_^2^) + (0.0612*P*)^2^ + 0.2027*P*] where *P* = (*F*_o_^2^ + 2*F*_c_^2^)/3
*S* = 1.02	(Δ/σ)_max_ < 0.001
3348 reflections	Δρ_max_ = 0.30 e Å^−^^3^
184 parameters	Δρ_min_ = −0.20 e Å^−^^3^
Primary atom site location: structure-invariant direct methods	Extinction correction: none

### Special details

**Table:**

Geometry. All e.s.d.'s (except the e.s.d. in the dihedral angle between two l.s. planes) are estimated using the full covariance matrix. The cell e.s.d.'s are taken into account individually in the estimation of e.s.d.'s in distances, angles and torsion angles; correlations between e.s.d.'s in cell parameters are only used when they are defined by crystal symmetry. An approximate (isotropic) treatment of cell e.s.d.'s is used for estimating e.s.d.'s involving l.s. planes.
Refinement. Refinement of *F*^2^ against ALL reflections. The weighted *R*-factor *wR* and goodness of fit *S* are based on *F*^2^, conventional *R*-factors *R* are based on *F*, with *F* set to zero for negative *F*^2^. The threshold expression of *F*^2^ > σ(*F*^2^) is used only for calculating *R*-factors(gt) *etc*. and is not relevant to the choice of reflections for refinement. *R*-factors based on *F*^2^ are statistically about twice as large as those based on *F*, and *R*- factors based on ALL data will be even larger.

### Fractional atomic coordinates and isotropic or equivalent isotropic displacement parameters (Å^2^)

**Table:**

	*x*	*y*	*z*	*U*_iso_*/*U*_eq_	
S1	0.14427 (8)	0.00747 (7)	0.26949 (6)	0.0589 (2)	
O3	0.02375 (19)	−0.13695 (17)	0.32049 (15)	0.0545 (4)	
C1	0.2907 (3)	−0.0764 (2)	0.1975 (2)	0.0494 (5)	
O4	0.0552 (2)	−0.41635 (18)	0.23259 (14)	0.0597 (4)	
C13	0.1215 (3)	−0.3741 (2)	0.3550 (2)	0.0460 (5)	
C12	0.2000 (3)	−0.4645 (3)	0.4347 (2)	0.0538 (6)	
H12	0.2116	−0.5631	0.4044	0.065*	
O1	0.0236 (3)	0.0562 (2)	0.17935 (19)	0.0802 (6)	
C8	0.1067 (3)	−0.2266 (2)	0.4028 (2)	0.0475 (5)	
O2	0.2394 (2)	0.11439 (19)	0.37184 (19)	0.0777 (6)	
C6	0.4487 (3)	−0.0852 (3)	0.2655 (2)	0.0572 (6)	
H6	0.4773	−0.0476	0.3492	0.069*	
C5	0.5639 (3)	−0.1506 (3)	0.2077 (2)	0.0616 (6)	
H5	0.6705	−0.1567	0.2533	0.074*	
C9	0.1631 (3)	−0.1736 (3)	0.5253 (2)	0.0626 (7)	
H9	0.1489	−0.0762	0.5561	0.075*	
C11	0.2616 (3)	−0.4110 (3)	0.5584 (2)	0.0629 (7)	
C2	0.2475 (3)	−0.1326 (3)	0.0737 (2)	0.0637 (7)	
H2	0.1408	−0.1269	0.0280	0.076*	
C4	0.5240 (3)	−0.2066 (3)	0.0845 (3)	0.0608 (6)	
C10	0.2412 (4)	−0.2652 (4)	0.6028 (2)	0.0706 (7)	
H10	0.2808	−0.2287	0.6862	0.085*	
C3	0.3654 (4)	−0.1975 (3)	0.0186 (3)	0.0726 (8)	
H3	0.3368	−0.2358	−0.0650	0.087*	
C7	0.6524 (4)	−0.2766 (4)	0.0219 (3)	0.0907 (10)	
H7A	0.6063	−0.3878	0.0013	0.136*	
H7B	0.7620	−0.2589	0.0773	0.136*	
H7C	0.6702	−0.2274	−0.0526	0.136*	
C14	0.3479 (4)	−0.5125 (4)	0.6429 (3)	0.0940 (10)	
H14A	0.4638	−0.4553	0.6794	0.141*	
H14B	0.3544	−0.6069	0.5955	0.141*	
H14C	0.2802	−0.5397	0.7075	0.141*	
C15	0.0926 (5)	−0.5547 (4)	0.1778 (3)	0.0943 (10)	
H15A	0.2171	−0.5422	0.1858	0.141*	
H15B	0.0416	−0.5717	0.0913	0.141*	
H15C	0.0444	−0.6435	0.2191	0.141*	

### Atomic displacement parameters (Å^2^)

**Table:**

	*U*^11^	*U*^22^	*U*^33^	*U*^12^	*U*^13^	*U*^23^
S1	0.0625 (4)	0.0381 (3)	0.0820 (5)	0.0201 (3)	0.0184 (3)	0.0062 (3)
O3	0.0500 (9)	0.0452 (8)	0.0746 (11)	0.0201 (7)	0.0160 (8)	0.0087 (7)
C1	0.0540 (13)	0.0374 (11)	0.0601 (14)	0.0134 (10)	0.0137 (11)	0.0086 (10)
O4	0.0777 (11)	0.0461 (9)	0.0574 (10)	0.0216 (8)	0.0090 (8)	0.0003 (7)
C13	0.0429 (11)	0.0438 (11)	0.0540 (13)	0.0108 (9)	0.0157 (10)	0.0054 (10)
C12	0.0507 (13)	0.0521 (13)	0.0676 (16)	0.0201 (11)	0.0225 (12)	0.0156 (11)
O1	0.0841 (13)	0.0644 (11)	0.1083 (15)	0.0442 (10)	0.0178 (11)	0.0297 (11)
C8	0.0453 (12)	0.0447 (11)	0.0562 (14)	0.0143 (9)	0.0138 (10)	0.0058 (10)
O2	0.0839 (13)	0.0459 (9)	0.1019 (14)	0.0120 (9)	0.0253 (11)	−0.0149 (9)
C6	0.0562 (14)	0.0547 (14)	0.0599 (15)	0.0118 (11)	0.0095 (12)	0.0019 (11)
C5	0.0524 (14)	0.0618 (15)	0.0749 (17)	0.0186 (12)	0.0144 (12)	0.0111 (13)
C9	0.0694 (16)	0.0578 (14)	0.0625 (16)	0.0150 (13)	0.0202 (13)	−0.0029 (12)
C11	0.0477 (13)	0.0779 (18)	0.0681 (17)	0.0152 (12)	0.0168 (12)	0.0248 (14)
C2	0.0634 (15)	0.0665 (16)	0.0623 (16)	0.0202 (13)	0.0043 (12)	0.0113 (13)
C4	0.0647 (16)	0.0496 (13)	0.0744 (17)	0.0145 (12)	0.0282 (13)	0.0106 (12)
C10	0.0700 (17)	0.0835 (19)	0.0542 (15)	0.0089 (15)	0.0104 (13)	0.0046 (14)
C3	0.090 (2)	0.0742 (18)	0.0568 (16)	0.0194 (16)	0.0220 (15)	0.0027 (13)
C7	0.093 (2)	0.080 (2)	0.114 (3)	0.0278 (18)	0.056 (2)	0.0064 (18)
C14	0.0724 (19)	0.129 (3)	0.092 (2)	0.036 (2)	0.0139 (17)	0.052 (2)
C15	0.144 (3)	0.0675 (18)	0.075 (2)	0.045 (2)	0.0110 (19)	−0.0157 (15)

### Geometric parameters (Å, °)

**Table:**

S1—O2	1.4154 (19)	C9—H9	0.9300
S1—O1	1.4219 (19)	C11—C10	1.383 (4)
S1—O3	1.5954 (17)	C11—C14	1.514 (4)
S1—C1	1.751 (2)	C2—C3	1.382 (4)
O3—C8	1.414 (3)	C2—H2	0.9300
C1—C2	1.376 (3)	C4—C3	1.376 (4)
C1—C6	1.380 (3)	C4—C7	1.516 (3)
O4—C13	1.360 (3)	C10—H10	0.9300
O4—C15	1.421 (3)	C3—H3	0.9300
C13—C12	1.383 (3)	C7—H7A	0.9600
C13—C8	1.393 (3)	C7—H7B	0.9600
C12—C11	1.382 (3)	C7—H7C	0.9600
C12—H12	0.9300	C14—H14A	0.9600
C8—C9	1.364 (3)	C14—H14B	0.9600
C6—C5	1.381 (3)	C14—H14C	0.9600
C6—H6	0.9300	C15—H15A	0.9600
C5—C4	1.369 (4)	C15—H15B	0.9600
C5—H5	0.9300	C15—H15C	0.9600
C9—C10	1.376 (4)		
			
O2—S1—O1	120.05 (12)	C10—C11—C14	121.2 (3)
O2—S1—O3	108.67 (11)	C1—C2—C3	118.8 (2)
O1—S1—O3	102.79 (10)	C1—C2—H2	120.6
O2—S1—C1	108.96 (11)	C3—C2—H2	120.6
O1—S1—C1	110.55 (12)	C5—C4—C3	118.6 (2)
O3—S1—C1	104.58 (9)	C5—C4—C7	120.7 (3)
C8—O3—S1	117.90 (13)	C3—C4—C7	120.7 (3)
C2—C1—C6	120.6 (2)	C9—C10—C11	120.9 (2)
C2—C1—S1	119.77 (18)	C9—C10—H10	119.6
C6—C1—S1	119.66 (18)	C11—C10—H10	119.6
C13—O4—C15	116.8 (2)	C4—C3—C2	121.5 (2)
O4—C13—C12	125.8 (2)	C4—C3—H3	119.2
O4—C13—C8	116.1 (2)	C2—C3—H3	119.2
C12—C13—C8	118.1 (2)	C4—C7—H7A	109.5
C11—C12—C13	121.2 (2)	C4—C7—H7B	109.5
C11—C12—H12	119.4	H7A—C7—H7B	109.5
C13—C12—H12	119.4	C4—C7—H7C	109.5
C9—C8—C13	121.4 (2)	H7A—C7—H7C	109.5
C9—C8—O3	121.2 (2)	H7B—C7—H7C	109.5
C13—C8—O3	117.30 (19)	C11—C14—H14A	109.5
C1—C6—C5	119.3 (2)	C11—C14—H14B	109.5
C1—C6—H6	120.4	H14A—C14—H14B	109.5
C5—C6—H6	120.4	C11—C14—H14C	109.5
C4—C5—C6	121.2 (2)	H14A—C14—H14C	109.5
C4—C5—H5	119.4	H14B—C14—H14C	109.5
C6—C5—H5	119.4	O4—C15—H15A	109.5
C8—C9—C10	119.5 (2)	O4—C15—H15B	109.5
C8—C9—H9	120.3	H15A—C15—H15B	109.5
C10—C9—H9	120.3	O4—C15—H15C	109.5
C12—C11—C10	118.9 (2)	H15A—C15—H15C	109.5
C12—C11—C14	119.9 (3)	H15B—C15—H15C	109.5
			
O2—S1—O3—C8	−59.07 (17)	S1—O3—C8—C13	−104.3 (2)
O1—S1—O3—C8	172.70 (15)	C2—C1—C6—C5	0.3 (4)
C1—S1—O3—C8	57.18 (17)	S1—C1—C6—C5	−179.55 (18)
O2—S1—C1—C2	−154.40 (19)	C1—C6—C5—C4	0.0 (4)
O1—S1—C1—C2	−20.4 (2)	C13—C8—C9—C10	2.0 (4)
O3—S1—C1—C2	89.5 (2)	O3—C8—C9—C10	179.2 (2)
O2—S1—C1—C6	25.4 (2)	C13—C12—C11—C10	1.0 (4)
O1—S1—C1—C6	159.38 (18)	C13—C12—C11—C14	−179.6 (2)
O3—S1—C1—C6	−90.64 (19)	C6—C1—C2—C3	−0.2 (4)
C15—O4—C13—C12	−9.7 (3)	S1—C1—C2—C3	179.6 (2)
C15—O4—C13—C8	170.7 (2)	C6—C5—C4—C3	−0.4 (4)
O4—C13—C12—C11	−179.3 (2)	C6—C5—C4—C7	179.4 (2)
C8—C13—C12—C11	0.3 (3)	C8—C9—C10—C11	−0.7 (4)
O4—C13—C8—C9	177.8 (2)	C12—C11—C10—C9	−0.8 (4)
C12—C13—C8—C9	−1.8 (3)	C14—C11—C10—C9	179.8 (2)
O4—C13—C8—O3	0.6 (3)	C5—C4—C3—C2	0.4 (4)
C12—C13—C8—O3	−179.08 (18)	C7—C4—C3—C2	−179.3 (3)
S1—O3—C8—C9	78.4 (2)	C1—C2—C3—C4	−0.2 (4)

### Hydrogen-bond geometry (Å, °)

**Table:**

*D*—H···*A*	*D*—H	H···*A*	*D*···*A*	*D*—H···*A*
C15—H15C···O1^i^	0.96	2.60	3.327 (4)	133

Symmetry codes: (i) *x*, *y*−1, *z*.
